# 

**Published:** 1915-10-02

**Authors:** 


### Buildings Contemplated.

Ennis.—Tenders are invited for the erection of new dormitories at the asylum at Ennis. Messrs. Sheehy and Tierney, 57 George Street, Limerick, are the architects.

Rochdale.—The Local Government Board have declined for the present to give their sanction to the loan for the purchase of the Springfield estate and the erection and equipment of a sanatorium for consumptives. The estate alone will cost £13,500.

Rates of Subscription: Twelve months, 8/8; Six months, 4/4; Three months, 2/2, post free to any address in the United Kingdom. Abroad, Twelve months, 8/8; Six months, 5/-; Three months, 2/6, post free.—Address The Subscription Dept., "The Hospital," The Hospital Building, 28/29 Southampton Street, Strand, London, W.C.

